# Risk of invasion and disease transmission by the Australasian freshwater snail *Orientogalba viridis* (Lymnaeidae): a field and experimental study

**DOI:** 10.1186/s13071-024-06403-5

**Published:** 2024-07-27

**Authors:** Antonio A. Vázquez, Elodie Chapuis, Jorge Sánchez, Pilar Alda, Dominique Faugère, Mónica Sánchez, Léa Souq, Joaquín López-Soriano, Sergio Quiñonero-Salgado, Nicolás Bonel, Jean-Pierre Pointier, Annia Alba, Sylvie Hurtrez-Boussès

**Affiliations:** 1 IHPE, UMR 5244 Université de Perpignan Via Domitia, CNRS, IFREMER, Université de Montpellier, Perpignan, France; 2 Laboratorio de Malacología, Instituto de Medicina Tropical Pedro Kourí, Havana, Cuba; 3 MIVEGEC, University of Montpellier, CNRS, IRD, Montpellier, France; 4 CREES, Montpellier, France; 5 Genética y Ecología Evolutiva, CERZOS, CONICET-UNS, Bahía Blanca, Buenos Aires Argentina; 6 Associació Catalana de Malacologia, Museu Blau, Barcelona, Spain; 7 PSL Research University, UAR 3278 CNRS-EPHE, CRIOBE Université de Perpignan, Perpignan, France; 8 Institut ExposUM, Université de Montpellier, Montpellier, France; 9 Département de Biologie-Écologie, Faculté des Sciences, Université de Montpellier, Montpellier, France

**Keywords:** Biological invasions, Parasite transmission, Demography, Lymnaeidae, Fasciolosis

## Abstract

**Background:**

Biological invasions pose risks to the normal functioning of ecosystems by altering the structure and composition of several communities. Molluscs stand out as an extensively studied group given their long history of introduction by either natural or anthropogenic dispersal events. An alien population of the lymnaeid species *Orientogalba viridis* was first sighted in 2009 in southern Spain. In its native range (Australasian), this species is one of the main intermediate hosts of *Fasciola hepatica*, a major worldwide trematode parasite largely affecting humans, domestic animals and wildlife.

**Methods:**

We collected field populations of *O. viridis* from its native (Malaysia) and invaded (Spain) ranges. We performed detailed morphoanatomical drawings of the species and screened for natural infection of parasites. Individuals were molecularly characterized using ITS2 for comparison with existing sequences in a fine phylogeography study. We founded experimental populations at two different conditions (tropical, 26 °C and temperate, 21 °C) to study the life-history traits of exposed and non-exposed individuals to different *F. hepatica* isolates.

**Results:**

We found a 9% natural prevalence of trematode infection (98% similarity with a sequence of *Hypoderaeum conoideum* [Echinostomatidae]) in the Spanish field population. The haplotypes of *O. viridis* found in our study from Spain clustered with Australian haplotypes. Experimental infection with *F. hepatica* was successful in both experimental conditions but higher in tropical (87% prevalence) than in temperate (73%). Overall lifespan, however, was higher in temperate conditions (mean 32.5 ± 7.4 weeks versus 23.3 ± 6.5) and survivorship remained above 70% during the first 20 weeks. In parasite-exposed populations, life expectancy dropped from an overall 37.75 weeks to 11.35 weeks but still doubled the time for initial cercariae shedding. Cercariae shedding started at day 23 post-exposure and peaked between days 53 and 67 with an average of 106 metacercariae per snail.

**Conclusions:**

Whether *O. viridis* will succeed in Europe is unknown, but the odds are for a scenario in which a major snail host of *F. hepatica* occupy all available habitats of potential transmission foci, ravelling the epidemiology of fasciolosis. This research provides a comprehensive understanding of *O. viridis* biology, interactions with parasites and potential implications for disease transmission dynamics, offering valuable insights for further research and surveillance.

**Graphical Abstract:**

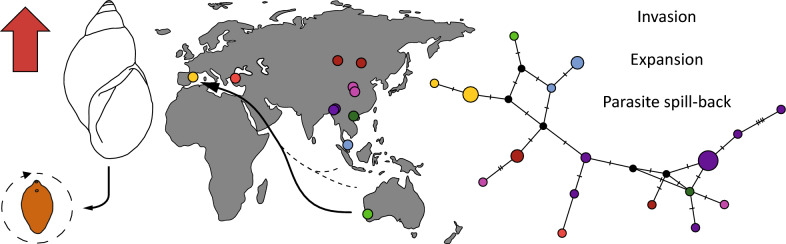

**Supplementary Information:**

The online version contains supplementary material available at 10.1186/s13071-024-06403-5.

## Background

Biological invasions represent pivotal events that pose risks to the normal functioning of ecosystems by altering the structure and composition of several plant and animal communities [1]. This is particularly serious in fragile freshwater habitats [2]. The arrival of alien species may not only disrupt the ecological equilibrium at the introduction site by altering trophic interactions, modifying resource availability and affecting community dynamics [3, 4] but also have a broader negative effect that sometimes threatens local public and veterinary health [5]. Literature on invasive species and disease ecology has shown the potential consequences of introducing non-native species to new environments [6]. For instance, the spread of West Nile or Dengue virus is often associated with the movement of mosquitoes such as the invasive species *Culex pipiens* or *Aedes albopictus* [7].

Within the category of freshwater invaders, molluscs stand out as an extensively studied group given their long history of introduction by either natural or anthropogenic dispersal events [8, 9]. The passive introduction of freshwater snails more frequently occurs through wading birds from near to medium range [10, 11]. However, human activities are usually the common way to mediate long-distance incidental introductions, typically related to the aquarium plant trade [12] or directly by deliberately introducing a particular snail species [13]. Invasions by snails notably influence the spread of infectious diseases since they transmit helminthic parasites [14]. For example, the prolific invasiveness of many lymnaeid species, commonly known as pond snails (Hygrophila: Lymnaeidae), has led to the emergence and re-emergence of fasciolosis – a globally neglected disease primarily transmitted by *Fasciola hepatica* to livestock, wildlife and humans [15]. Fasciolosis (transmitted mainly by *F. hepatica* and *Fasciola gigantica*) is a worldwide parasitic disease largely affecting cattle and humans in several countries [16]. These trematode parasites have a complex life cycle with a definitive host (mammal in which sexual reproduction occurs) and always a lymneid snail as intermediate host (where asexual reproduction takes place). Over 30 species of lymnaeids have been found naturally or experimentally to develop fasciolids (see for review [17]).

An illustrative example of how a non-native snail species can pose a threat to biodiversity and public health is the case of the lymnaeid species *Orientogalba viridis* (Quoy and Gaimard, 1832). In 2009, an alien population was observed in southern Spain for the first time [18]. Subsequently, stable populations were confirmed in several municipalities surrounding the delta of the Ebro River [19]. The lymnaeid *O. viridis* was first described from the island of Guam (Pacific Ocean, type locality) but it extends throughout Australasia from the Korean Peninsula to Australia and several Pacific islands [20]. In its native range, this species transmits fasciolosis, with a reported natural prevalence of 1.57% infection with *Fasciola gigantica* [21]. Previous experimental infections of *O. viridis* originating from Australia, Nepal, and South Korea with *F. hepatica* showed prevalence ranging between 49–64% [15]. After an initial misidentification far from its native range in Turkey (as *Radix* sp., see [22]), *O. viridis* arrived in Spain. As indicated in previous distribution studies, this species appears to tolerate a wide range of temperatures ranging from temperate Mongolia (from −10 °C to 25°C) to tropical Pacific islands (24–32 °C) [20]. The capacity of *O. viridis* to stand a wide range of temperatures makes it a significant threat to tropical and temperate exotic environments if introductions are successful as has been recently recorded in Lake Malawi, Africa [23].

**Fig. 1:**
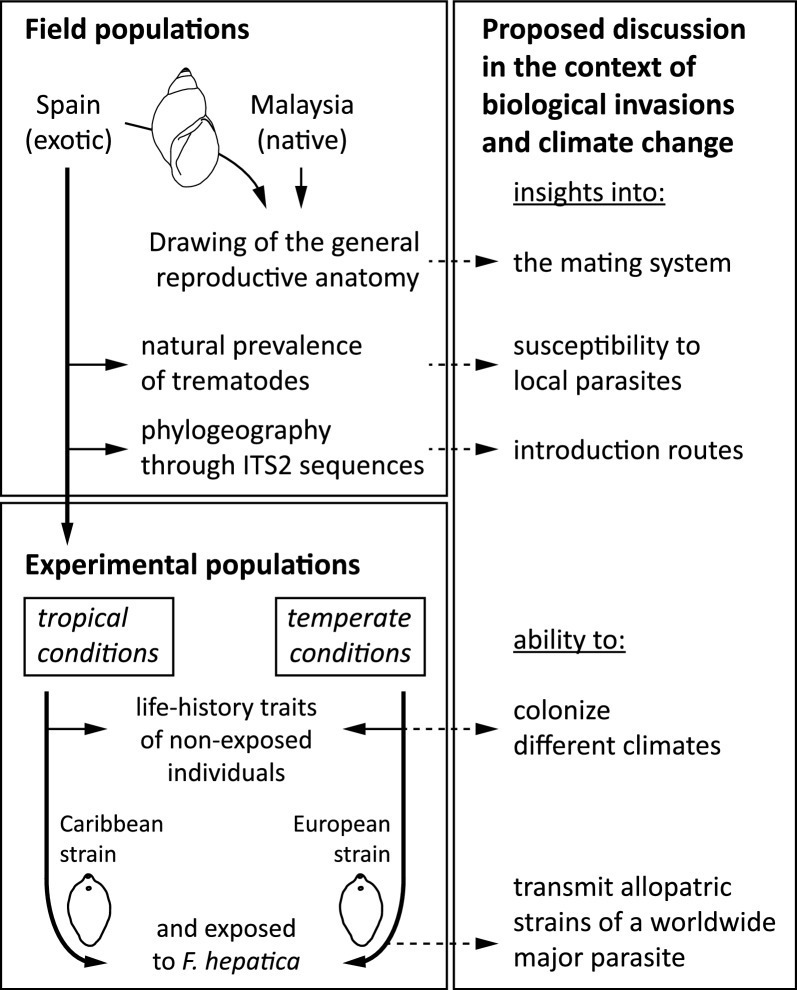
Methodological conception of the observational and experimental assessment of the introduced lymnaeid snail *Orientogalba viridis* in southern Europe

In this study, our objective is to analyse and discuss the risk of expansion of the snail species *O. viridis* and *F. hepatica*, which may threaten local fauna and enhance disease transmission in humans and livestock. Our objectives align with those of the One Health initiative and explore the implications of climate change given that this species usually transmits fasciolosis in its native region (warmer conditions than southern Europe) [24]. Our assessment involves several key steps (refer to Fig. 1 for a simplified methodological conception). First, we comprehensively outlined and described the reproductive anatomy, comparing various traits between native and introduced field-collected individuals to explore the mating system of this snail and its potential implications for invasion success. Second, we dissected introduced field snails to investigate the presence of natural trematode infections. Third, we conducted a phylogeographic analysis using 25 ITS2 sequences from both native and non-native regions to gain insights into the species distribution. Finally, we experimentally analysed the demographic patterns and infection capacity of this species at different temperatures, aiming to discuss the risks posed by the species and the potential spillback effect of the parasite in Europe and western tropical countries.

## Methods

### Study organism and field sampling

**Fig. 2:**
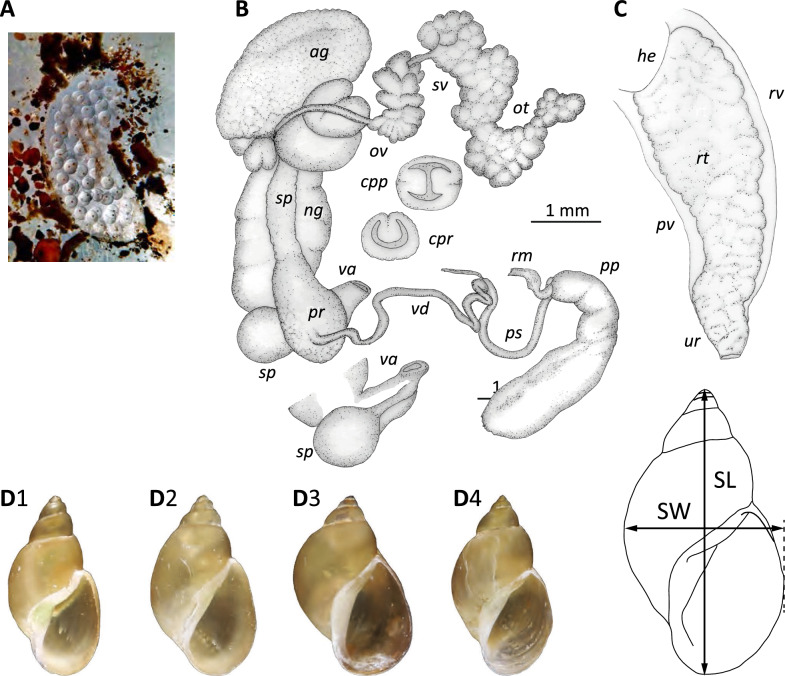
Shell morphology and internal anatomy of *Orientogalba viridis* from introduced populations in Deltebre, Spain. **A** Newly laid egg mass of *O. virdis* (51 eggs). **B** Complete reproductive system anatomy (*ag* albumen gland, *cpp* cross-section of the preputium, *cpp* cross-section of the prostate, *ng* nidamental gland, *ot* ovotestis, *ov* oviduct, *pp* preputium, *pr* prostate, *ps* penis sheath, *rm* retractor muscle, *sp* spermatheca, *sv* seminal vesicle, *va* vagina, *vd* vas deferens). **C** Anatomy of the kidney (*he* heart, *pv* pulmonary vein, *rt* renal tube, *rv* renal vein, *ur* ureter). **D** Variation in shell shapes (D1, 8 mm; D2, 8.2 mm; D3, 9.3 mm; D4, 11.9 mm)

*Orientogalba viridis* (Gastropoda: Lymnaeidae) is a freshwater snail native to the Australasian region [20]. The species inhabits various freshwater environments, including ponds, flooded crops and slow-moving streams. Typically, associated with aquatic vegetation, *O. viridis* primarily feeds on algae, detritus and other plant material found in their habitats [15]. Like all freshwater pulmonates, *O. viridis* is hermaphroditic and capable of self-fertilization and cross-fertilization, providing advantages for invasion, particularly when it is introduced in small numbers; although, its exact mating strategy is not well studied. The snails lay eggs in clusters, forming egg masses attached to environmental substrata (Fig. 2C).

We collected adult snails from non-native (Spain) and native (Malaysia) populations. The non-native snails were gathered near l’Aldea (40.7312° N; 0.6323° E), Ebro Delta, Spain, in October 2020 along street’s sideway canals surrounded by rice paddies. The snails were transported alive (*n* > 100) to the laboratory for rearing. In the laboratory, these wild-caught snails were subdivided into four groups, kept in small plastic aquaria (430 cm^3^) at a stable temperature (23 ± 1 °C) with a 12–12 h photo-period and fed with algae ad libitum [25]. The native snails were collected in 2008 and fixed in 70% EtOH at Cameron in Malaysia (4.50756° N; 101.40728° E) from an artificial garden selling aquatic plants. For further analyses, ten individuals from each native and non-native population were subjected to immersion in water heated at 70 °C for 40 s, followed by transfer to water at room temperature. Next, the soft parts were drawn from the shell with small forceps applied to the cephalopedal mass and fixed in 70% ethanol. These 20 individuals were used for studying the internal anatomy and analysing the phylogeography of *O. viridis* (section 2.4). The remaining non-native snails that were transported to the laboratory were used to investigate natural trematode infections (section 2.3) and to perform experimental studies (sections 2.5 and 2.6).

### Anatomy of field populations of *O. viridis*

To elucidate the mating system contributing to snail invasion success, we conducted a detailed description of the reproductive system in individuals from both non-native and native populations. The tissue of each individual (*n* = 10, adults of 9–10 mm) was dissected under a stereoscopic microscope, and drawings of the reproductive system and kidney were made using a camera lucida attachment. We measured common reproductive anatomical traits used to discriminate lymnaeid snail species [26]: the prostate perimeter (PP) and surface (PS), penis sheath length (PsL) and preputium length (PL) using Motic Images Plus v.3.0.11.38 (Motic China Group Co., Ltd. 2015). The ratios of PP/PS and PsL/PL were then compared between populations.

### Parasitological assessment of naturally infected field individuals

To assess the presence of natural trematode infections in *O. viridis*, we dissected wild-caught snails (*n* = 56) from l’Aldea under a stereomicroscope following the methodology described in [27]. The visceral mass of infected snails exhibiting trematode larvae was preserved separately from the snail foot. DNA from the infected snail tissue was extracted using the DNeasy Blood and Tissue Kit (Qiagen) following the manufacturer’s instructions. Polymerase chain reaction (PCR) with trematode-specific primers targeting the 16S gene was conducted: TREM_16_F1 5′-GACGGAAAGACCCCRAGA-3′ (forward) and TREM_16_R2 5′-CRCCGGTYTTAACTCARYTCAT-3′ (reverse) [28]. The DNA was amplified in a total volume of 25 μl, including 5 μl of buffer (5×), 3 μl of MgCl2 (25 mM), 1.25 μl of dNTPs (2.5 mM), 0.5 μl of each primer (1:10; reverse and forward), 0.5 μl of Taq DNA polymerase and 2.5 μl of DNA. The amplification process involved an initial denaturation step at 94 °C for 3 min, followed by 40 cycles of denaturation at 95 °C for 30 s, annealing at 54 °C for 30 s, extension at 72 °C for 15 s and a final extension step at 72 °C for 5 min [28]. Amplicons were visualized in 2% agarose gels stained with EZ-Vision and subsequently sent for DNA sequencing by Eurofins Genomics (Ebersberg, Germany) using the same primer set. The sequences were uploaded to GenBank (accession number: OR754222), and parasite identity was determined using n-BLAST (https://blast.ncbi.nlm.nih.gov/Blast.cgi).

### Phylogeographic analysis of *O. viridis*

To explore the phylogeographic relationships of native and non-native populations and to explore the ancestral geographic state of the species, we extracted DNA from the foot of randomly selected Spanish individuals (*n* = 2) and Malaysian individuals (*n* = 3). The Chelex protocol, as described previously [29], was used for DNA extraction. The internal transcribed spacer 2 (ITS2) gene was amplified using the primers NEWS2 (forward) 5′ TGTGTCGATGAA-GAACGCAG 3′ and ITS2-RIXO (reverse) 5′ TTCTATGCTTAAATT-CAGGGG 3′ [30]. PCR amplification was performed in a total volume of 25 μl containing 5 μl of buffer (5X), 3 μl of MgCl2 (25 mM), 1.25 μl of dNTPs (2.5 mM), 0.5 μl of each primer (2 μM; reverse and forward), 0.5 μl of Taq DNA polymerase and 2.5 μl of DNA (dilution 1/100) in a thermal cycler. The snail DNA amplification consisted of an initial denaturation step at 95 °C for 15 min; 35 cycles of denaturation at 94 °C for 30 s, annealing at 52 °C for 90 s, and extension at 72 °C for one min; and a final elongation at 60 °C for 30 min.

The presence and size of the amplification products of snail DNA were confirmed through electrophoresis on 2% agarose gels stained with EZ-Vision. DNA sequencing was performed by Eurofins Genomics (Ebersberg, Germany) using PCR-amplified products as templates. The sequences were uploaded to GenBank (OR728031-OR728032 from Spain and OR728033–OR728035 from Malaysia) and analysed for snail phylogeography. A search in the literature and GenBank was performed for sequence data of the ITS2 gene apparently attributable to lymnaeids of the species *O. viridis* (including the nomina “*Austropeplea viridis*” and “*Lymnaea viridis*” in the search). Coordinates were provided for most sites by the authors. We inferred coordinates from the locality data using Google Earth. Phylogeographic analyses were conducted on the five sequences obtained in this study, along with 20 other sequences retrieved from GenBank (Supplementary Table 1). Alignments were performed individually for each gene using MAFFT [31]. Ambiguously aligned sites were excluded using GBLOCKS with default settings for less stringent selection [32]. The final sequences comprised 371 positions (68% of the original 539 positions). A TCS network was constructed using PopART [33]. The map was created in QGIS (https://www.qgis.org), vectors were downloaded from https://www.naturalearthdata.com/, and figures were edited in GIMP (https://www.gimp.org).

Bayesian inference in Beast2 [34] was employed to assess the ancestral geographical state of the species offer a systematic approach to combining geographic and genetic data [35]. The best-fitting models of sequence evolution for each gene were selected using bModelTest [36]. The best model describing the evolution was 123,145 + G + I. The analyses were run using four gamma categories and a proportion of 0.5 invariant sites. Uncorrelated relaxed-clock models were chosen for all loci. The relative clock mean priors were all lognormal (*M* = 0, *S* = 1). We used a birth–death model as a prior with lognormal birth and death rates. The country of each sequence was incorporated as a discrete trait. The MCMC was run for 200,000,000 generations, storing every 20,000 generations. The MCMC output was visualized using Tracer [37], and tree samples were summarized by TreeAnnotator (utility program distributed with the Beast package) using a 10% burn-in. The species tree was visualized and edited in FigTree (http://tree.bio.ed.ac.uk/software/figtree/) and GIMP (https://www.gimp.org).

### Demographic patterns and infection capacity at different temperatures

To establish experimental lines, we collected eggs from all wild-caught adults from the invaded spot in l’Aldea, Spain. After allowing the eggs to hatch for approximately 2 weeks, we combined all the juveniles into an aquarium and randomly selected 100 individuals. These snails constituted the first laboratory generation (G_1_) and acted as founders for the next generation (G_2_), initiating a new cycle. We used the second laboratory generation to set up two different experimental treatments: tropical (26 ± 1 °C) and temperate (21 ± 1 °C) conditions; the photoperiod and feeding conditions were the same. The details of this experiment are described in the following subsections.

We conducted two independent experimental assays to assess and compare the life history traits of the second laboratory generation of snails (G_2_) under different temperature conditions (tropical and temperate) and with or without exposure to parasitic infection (*n* = 30 snails per experimental group). Age intervals (*x*) were set up at 1 week, and snails were followed from hatching until the whole population expired. The following demographic parameters were measured at each age interval [38]: proportion of living individuals (*l*_x_), survival probability (*p*_x_), life expectancy (*e*_x_), fecundity rate (*m*_x_), hatching probability (*H*_x_), net reproduction rate (*R*_0_) and natural (*r*_x_) and finite (*λ*_x_) increase rates. Individual growth was estimated using the total length of the shell (SL) and the shell width measured with a calliper (0.01 mm), as illustrated in Fig. 2.

To perform the infection trials, we retrieved isolates of the liver fluke *F. hepatica* from newly hatched miracidia (infective larval stage) from eggs occurring in the bile of infected cattle from western Cuba (tropical assay) and from the central–northern region of Corsica (temperate assay). The used strains of *F. hepatica* should have a priori a different genetic background (not tested here) but would be adapted to transmit in two different climatic conditions. We were unfortunately unable to obtain local Spanish *F. hepatica.* In all instances, veterinary authorities granted permission to collect samples of bile from the gallbladders of infected cattle in accordance with current hygiene and best practice guidelines at local abattoirs. Bile examination and *F. hepatica* egg storage were conducted as described by Alba et al*.* [39], and experimental infections were performed as described by Vázquez et al*.* [40]. The infection trial involved exposing randomly selected 2-week-old individuals of *O. viridis*–G2 (*n* = 30) of both tropical (26–27 °C) and temperate (21–22 °C) temperature conditions to five miracidia (parasite dose). Snails reared at tropical conditions were exposed to Cuban *F. hepatica* while those reared at temperate conditions were exposed to Corsican *F. hepatica*. All exposed snails were dissected on day 25 post-exposure (d.p.e.) to verify infection [41]. Individuals who died before 25 d.p.e. were also dissected to detect any traces of *F. hepatica* larvae. We recorded the prevalence, parasite intensity (i.e., the number of rediae per snail) and overall survival of the snails following Reiczigel et al*.* [42].

### Dynamics of cercariae shedding and overall metacercariae production in *O. viridis* exposed to *F. hepatica* under tropical conditions

We used another group of G_2_ snails (*n* = 30) from a tropical assay to assess total metacercariae production in *O. viridis* and to explore the dynamics of cercariae shedding in this species. The same infection protocol was applied (section 2.5). At 15 d.p.e. (at least 10 days before cercariae shedding might occur), individuals were isolated in small plastic Petri dishes and kept under the same rearing conditions to measure the individual production of metacercariae. Dying snails were dissected daily, and the number of rediae was also reported at 1-day intervals. We reported metacercariae/snails/day from the first emission until the last emitting snail by counting all encysted metacercariae. The snails were transferred to a new Petri dish every day.

### Data analysis

All variables were initially assessed for a normal distribution (Shapiro–Wilk test) and variance homogeneity (Levene test). We used a Student’s *t*-test to compare anatomical indexes as well as to compare shell length and parasite intensities during infection trials. Proportion tests were used to compare the number of surviving snails, overall prevalence and the number of infected dead snails. Snail’s age and the number of rediae was associated through Spearman’s correlation coefficient. All statistical tests and fitting of parasitological and morphometric data were performed in Statistica v.12 (StatSoft Inc., Tulsa, OK, 2014). Life-history traits were adjusted to the best-fit curve using regression coefficients. Survival in each trial was analysed through Kaplan‒Meier curves in GraphPad Prism v.9.0.2 (GraphPad Software, LLC, 2021) and compared using a log-rank test. We reported mean values followed by 95% confidence interval (CI). For prevalence, we calculated CI using the score method [43]. Differences were considered statistically significant at values of *P* < 0.05.

## Results

### Comparative anatomy of the shell, reproductive and excretory system of *O. viridis* from Spain and Malaysia

The most prominent features of the reproductive system are as follows (Fig. 2B): (1) the spermiduct is flat, wide and ribbon shaped; (2) the prostate is voluminous and pear shaped; (3) the prostate invaginates into a fissure with a U-shaped appearance in cross-section; (4) the penis sheath is narrow and usually shorter than the preputium (overall PsL/PL < 1); (5) the preputium cross-section shows a two folds pattern, as observed in *Stagnicola elodes* and other lymnaeids [44]; and (6) the spermatheca is rounded with a short canal. The renal part is a flat, wide and straight tube that tapers distalward and ends at a slightly curved ureter (Fig. 2C). Overall, the shell has no spiral or axial sculpture on the body whorls, and the columellar edge is straight and presents no particular features, such as twist or plait. The body whorls are well rounded, and the sutures are well marked. We observed high variability (not measured) in overall shell shape (Fig. 2D). Concerning reproductive anatomy, individuals from l’Aldea showed a significantly (*P* < 0.001, Student’s *t*-test) lower prostate perimeter/surface ratio (PP/PS = 3.51 ± 0.34) than those from Cameron (PP/PS = 4.08 ± 0.20). We found no significant differences in the PsL/PL index between the populations (*P* = 0.24, Student’s *t*-test).

### Natural infection in the non-native snails

Dissection of field-generated individuals (*n* = 56) collected in Spain revealed a patent infection (8.93% prevalence, CI [3.87%–19.26%]) with rediae of an unknown trematode occurring in the gonad of the snails. The analysis of the trematodes infecting the snails using the 16S region showed 98% similarity with a sequence assigned to *Hypoderaeum conoideum* (Echinostomatidae) collected from a free-range duck in Hubei Province, China (accession number: KM111525; [45]). In addition, we observed an 85.7% (CI [74.3%–, 92.6%]) prevalence of *Chaetogaster* cf. *limnei* (Annelida: Naididae) living in the mantle cavity of the snails.

### Phylogeography of DNA-sequences of *O. viridis*

**Fig. 3:**
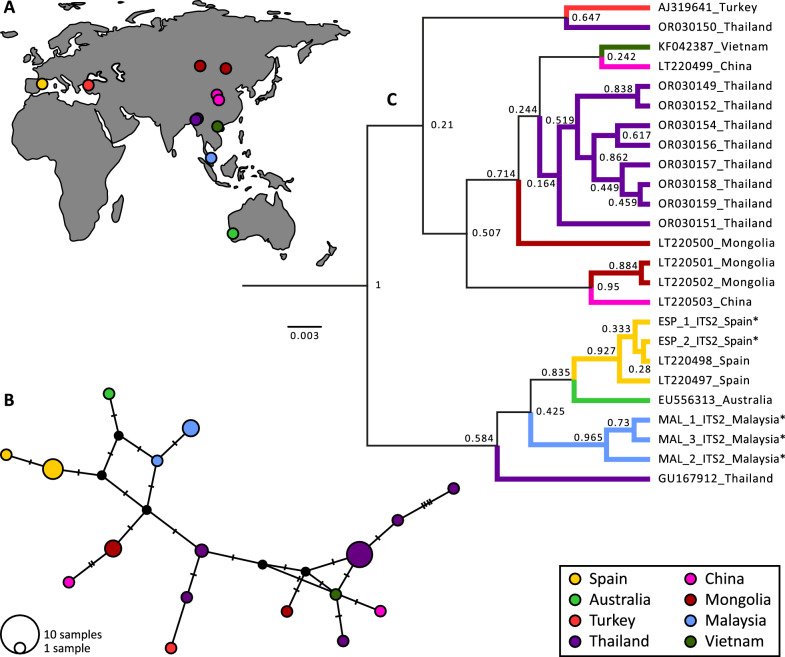
Phylogeographic relationships of existing ITS2 data on *Orientogalba viridis*. **A** Geographic distribution of populations used in the analysis. **B** Haplotype network, where circle sizes are proportional to haplotype frequencies and colours represent the respective sample regions in the map. **C** TCS tree (statistical parsimony) using 25 available ITS2 sequences of *O. viridis*. The haplotype found in this study clustered with an Australian haplotype and another Spanish haplotype from an individual of the same locality sampled in 2015 [18]

We uploaded five snail sequences to GenBank: two identical sequences from Spain and three (two identical) sequences from Malaysia. The Spanish haplotype found in this study is identical to that found in the same area (Deltebre) in 2015 [18]. The haplotype network showed that the Spanish haplotype found in this study clustered with the Australian haplotype and with the other Spanish haplotype (Fig. 3). The other haplotype from a non-native region, Turkey, clustered with the haplotypes from individuals collected in the overall native region of *O. viridis*. The Malaysian haplotypes found in this study (from field-collected individuals from Cameron) clustered within the native group. We found that Thailand might be the geographical origin of all studied individuals of *O. viridis,* with a 34% probability (Supplementary Table 2).

### Comparative demographic traits of exposed and not exposed *O. viridis* to *F. hepatica*

The experimental infection was successful in both infection settings but was significantly higher in tropical (86.7% prevalence, CI [70.3%–94.7%]) than in temperate (73.3% prevalence, CI [55.5%–85.8%]). We found a similar pattern in parasite intensities and shell length in infected individuals for both conditions. However, there were significant differences (Student’s *t*-test, *P* < 0.001) between the infected (smaller size) and control groups (larger size) regardless of the treatment (Table 1).

**Table 1:** Snail survival (%), shell length (SL) and experimental prevalence (%) and parasite intensity (p.i. is the number of rediae/snail) in *Orientogalba viridis* from l’Aldea (Delta del Ebro) exposed to two *Fasciola hepatica* (*Fh*) isolates at different temperatures (*CI* confidence intervals)

	Cuban *Fh* isolate – snails at 26–27 °C			French *Fh* isolate – snails at 21–22 °C			Between treatments
	Exposed	Control	*P*	Exposed	Control	*P*	*P*
Percent alive	96.6	100^*^	*P* > 0.1	66.6	100^*^	*P* < 0.01	*P*_exp_ < 0.01
Mean SL (mm)	9.8	11.2	*P* < 0.01	2.6	4.1	*P* = 0.03	*P*_exp_ < 0.001 *P*_con_ < 0.001
Prevalence (%)	86.6	–	–	73.3	–	-	*P*_exp_ < 0.01
Infected dead snails	0	–	–	4	–	–	*P*_exp_ = 0.04
Mean p.i. (95% CI)	38.5 (7.9)	–	–	20.6 (5.6)	–	–	*P*_exp_ < 0.01

The data were collected on day 25 post-exposure (*n* = 30 in all cases). *P* values were obtained after a Student’s *t*-test (shell length and parasite intensity, *P*_exp_ = exposed, *P*_con_ = control) and a proportion test (survival, prevalence and infected dead snails)

**Fig. 4:**
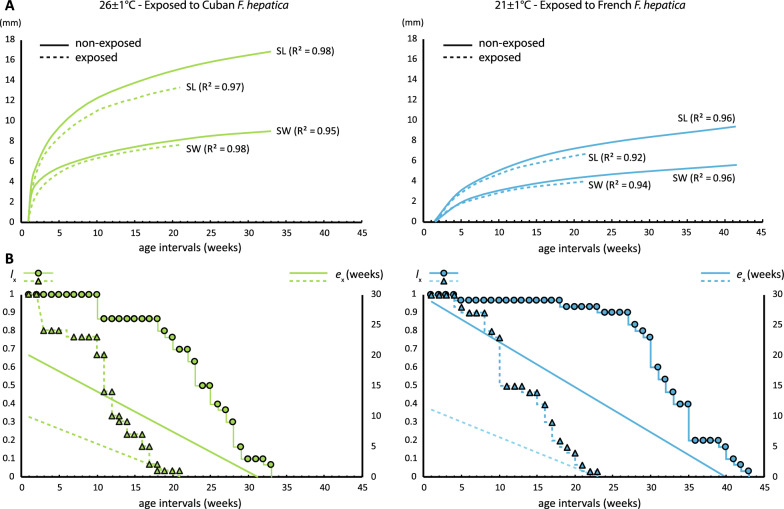
Growth and survival of experimental populations of *Orientogalba viridis* exposed and non-exposed to different *Fasciola hepatica* isolates (Cuban and French) under two different experimental conditions. **A** Adjusted (logarithmic) shell growth parameters (*SL* shell length, *SW* shell width, *AL* aperture length, *AW* aperture width, *SpL* spire length); **B** Survivorship (*l*_x_) and life expectancy (*e*_x_, linearly adjusted)

Individual growth from both treatments showed a rapid rate of increase in all measured parameters during the first 10 weeks. Then, a very slow rate of increase was maintained until death. All fitted curves had regression coefficients of *R*^2^ > 0.85 (Fig. 4A). Sexual maturity varied according to temperature settings and was reached after only 2 weeks in snails kept under tropical conditions but after 9 weeks in snails kept under temperate conditions. However, the overall lifespan was higher in temperate settings. In both experiments, survivorship was above 70% during the first 20 weeks, drastically dropping from week 22 under tropical conditions and week 31 under temperate conditions (Fig. 4B). Survivorship of the populations was significantly different between exposed and non-exposed individuals regardless of the conditions (log-rank test, *P* < 0.00001). The calculated life expectancy at birth for the non-exposed populations was 21.6 weeks (average lifespan 22.1 weeks) in tropical conditions and 29.5 weeks (average lifespan 31.8 weeks) in temperate conditions (Fig. 4B). However, in the exposed treatments, the life expectancy was 10.3 weeks (tropical) and 12.4 (temperate), but the life expectancy doubled the time needed for initial cercaria shedding (section 3.5).

**Fig. 5:**
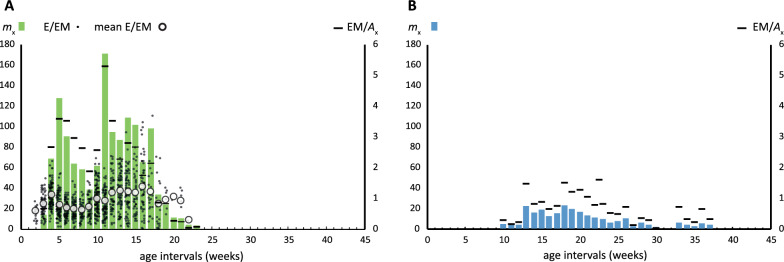
Reproductive traits of *Orientogalba viridis* reared under different conditions (*m*_x_ fecundity, *E* eggs, *EM* egg mass, *A*_x_ living individuals). **A** snails reared at 26 °C; **B** snails reared at 21 °C (note that we were unable to estimate E/EM in this experimental setting)

Reproductive traits were only assessed in the non-exposed experimental populations. Fecundity showed two peaks varying in time according to the experimental conditions (Fig. 5), but it was particularly lower under temperate conditions. The number of egg masses (EM) per individual alive (*A*_x_) increased during the first 10 weeks of reproductive activity and decreased towards the end of reproductive age. Overall EM/*A*_x_ mean ± CI in each condition was 2.05 ± 0.58 at 26 °C and 0.65 ± 0.15 at 21 °C. The overall hatching probability remained over 0.7 and peaked above 0.99 in the reproduction peaks (a parameter measured only under tropical conditions). The number of eggs (E) per egg mass varied significantly during reproductive age (overall range 2–112), with averages ± CI of 33.4 ± 3.8 and 33 ± 6.8during fecundity peaks at weeks 4 and 10, respectively (Fig. 5). However, a higher number of E/EM did not always translate to greater population increases due to a decrease in the number of EM led by each individual (Table 2). At the end of reproductive age, net reproduction differed between the two temperature treatments. The population under tropical conditions had a 6.4 times greater *R*_0_ than that under temperate conditions. At reproductive peaks, the population under tropical conditions also increased faster (8.3 times at first and 3.7 times at second reproductive peaks, respectively) than did that under temperate conditions. The finite rate of increase was, however, less different between conditions but followed the same pattern, being at least twice as high at both reproductive peaks.

**Table 2:** Summary of the life-history traits of the experimental populations of *Orientogalba viridis* reared under two different conditions at four different biological moments (age intervals in weeks, *x*; proportion of living individuals,* l*_x_; probability of survival,* p*_x_; life expectancy,* e*_x;_ hatching probability,* H*_x_; net reproduction rate,* R*_0_; natural, *r*_x_ and finite, *λ*_x_ increase rates)

	*x*	*l*_x_	*p*_x_	*e*_x_	*H*_x_	*R*_0_	*r*_x_	*λ*_x_
Experimental settings of 26 ± 1 °C								
Hatching	*x*_0_	1	1	21.6	-	0	0	0
Sexual maturity	*x*_2_	1	1	19.6	0.76	12	1.2	3.4
First reproductive peak	*x*_4_	1	1	17.6	0.99	204	1.5	4.5
Second reproductive peak	*x*_10_	0.87	1	13.6	0.99	643	1	2.7
Last reproduction	*x*_22_	0.5	0.73	4.7	0.68	1165	0.7	2.1
Experimental settings of 21 ± 1 °C								
Hatching	*x*_0_	1	1	29.5	–^*^	0	0	0
Sexual maturity	*x*_9_	0.97	1	23.2	–^*^	2.5	0.06	1.06
First reproductive peak	*x*_12_	0.97	1	20.2	–^*^	26	0.18	1.19
Second reproductive peak	*x*_17_	0.97	1	15.2	–^*^	100	0.27	1.31
Last reproduction	*x*_36_	0.2	1	4.3	–^*^	181	0.29	1.34

^*^H_x_ not measured in this experimental treatment

### Dynamics of cercariae shedding and overall metacercariae production in *O. viridis* exposed to *F. hepatica* under tropical conditions

**Fig. 6:**
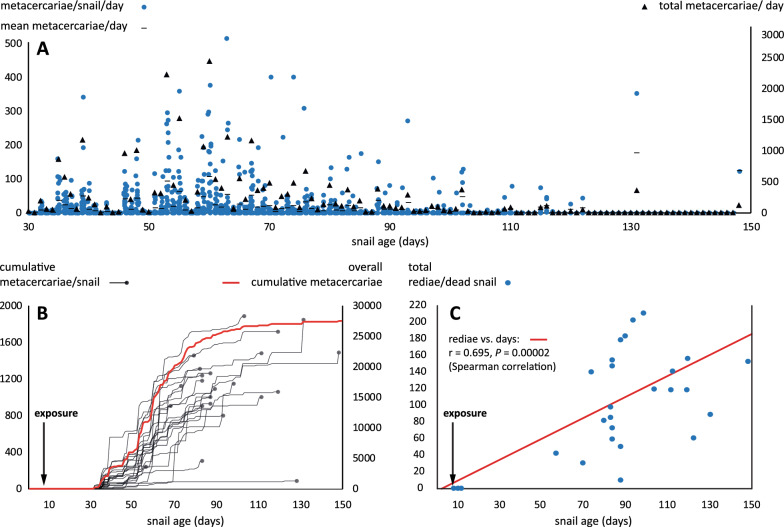
Chronobiology of parasite intensities in *Orientogalba viridis* reared at 26 °C (*n* = 30) exposed to Cuban *Fasciola hepatica* (5-miracidia/snail). **A** Daily metacercariae counts at the individual and population levels from the first day of emission (day 23 post-exposure) until the population expired. **B** Cumulative metacercariae production per snail (truncated lines indicate the death of the individual). **C** Parasite intensity (total rediae count) of dying individuals during the span of the experiment

Individuals (*n* = 30) exposed to five miracidia doses of *F. hepatica* were maintained until death, with the last snail dying at the age of 148 days. Cercariae shedding of isolated snails started on 23 d.p.e., with nine snails emitting an average of 2.33 ± 1.19 cercariae encysted into metacercariae (maximum of seven). We observed an emission peak between 53 and 67 d.p.e. (Fig. 6A). At 60 d.p.e., we reached a maximum count of 2434 metacercariae (average of 106 ± 44.6 metacercariae per snail). The highest number of metacercariae emitted by a single snail in one day was 516 at 63 d.p.e. The average number of daily metacercariae counted for the whole population was 303 ± 86.8, while the highest number of total metacercariae emitted by a single snail during its lifespan was 1888. Overall, when the population of *O. viridis* expired, 27,533 (918 per exposed snail) of the total amount of shed cercariae encysted into metacercariae (Fig. 6B).

We dissected all the snails that died throughout the experiment for rediae counting. Only six individuals who died during the first 7 d.p.e. failed to have observable parasitic larvae. However, we found a significant correlation (Spearman *r* = 0.695, *P* = 0.00002) between the age of the snail and the number of total rediae harboured (Fig. 6C). The greatest number of rediae in a single snail was 211 (mean 112.8 ± 54.5). Conversely, we failed to observe any correlation between the number of total counted metacercariae and the number of rediae observed at the time of death (Spearman *r* = 0.207, *P* = 0.2).

## Discussion

### A new snail host of *F. hepatica* settles in Europe

The first report of *O. viridis* in Europe (in Spain in 2009) [18] provided some insights into its internal anatomy, showing differences in the length of reproductive organs between native and exotic populations. However, little information has been presented concerning the relative length of the individual, the reproductive system and other organ features that would serve for comparison in this species. In this study, we produced for the first time a detailed schematic representation of the reproductive organs of several field-generated individuals. The general characteristics of the reproductive anatomy suggest that the species is a preferential outcrosser (large prostate and developed preputium and penis sheath) compared with other virtually selfing species within Lymnaeidae, such as *P. columella* (prostate is barely developed, [46]).

While selfer species are suggested to be better at founding new populations in introduced localities [47], *O. viridis* seems to have performed exceptionally well in establishing and colonizing a large portion of the Ebro delta [19]. There are examples of well-known preferential out-crosser species that have invaded large portions of the world, such as the freshwater snail *Physella acuta* [48] and the land snail *Lissachatina fulica* [49]. However, little information is available for these species at the very early stages of invasion regarding selfing and fecundity rates in the field. Understanding the reproductive strategies and anatomical features of invasive species is crucial for predicting and managing their spread and impact on ecosystems.

### From Southeast Asia to the world

In this study, the population from Spain clustered with those of Australia but not with the other introduced population in Turkey. Both introduced populations, however, appear to have similarities with analysed individuals from Thailand, suggesting a common original area but probably completely different introduction events. Individuals from Söke, Turkey were previously molecularly analysed by [22]; those from Deltebre (Spain) were first sighted in 2009 in scattered rice paddies [19]. A maximum-parsimony tree using ITS-2 of several sequences covering a vast part of the phylogeny of Lymnaeidae has been presented for Spanish *O. viridis* [18].

Here, we attempt to construct a refined tree using only available sequences with more than 95% similarity to those of Spanish individuals. While more localities should be sampled to better assess the origin of introduced populations out of the native range of the species (Australasian, see [20]), the epicentre of analysed sequences points to the region around Thailand. Freshwater snails are self-propelled species, but their expansion into allopatric or even parapatric distributions is more commonly associated with passive migration through hydro or zoochoria mechanisms. Long-distance dispersal, however, usually relies on wading birds or human translocations. Some renowned examples of human-mediated land and freshwater snail invasions are the giant African Snail (*Lissachatina fulica*) [50], the rosy wolfsnail (*Euglandina rosea*) [51], the New Zealand mud snail (*Potamopyrgus* antipodarum) [52] and the golden applesnail (*Pomacea canaliculata*) [53]. For instance, human activities are probably responsible for the flash invasions of freshwater snails in the last couple of centuries and has been particularly promoted by the intentional introduction of species through the aquarium pet trade [54].

Such activity is responsible for most of the patchy distributions observed today in many freshwater snail species worldwide. For instance, incidental introductions of lymnaeid species associated with the commerce of aquarium plants (see, for example, the case of *Pseudosuccinea columella* [54]) may result in an expansion of the populations in the introduced areas if conditions are suitable for the species [55, 56]. We cannot know whether *O. viridis* will successfully colonize the wetlands of the Mediterranean basin or farther tropical areas in the Caribbean or South America, but the introduced haplotype seems to perform very well under both conditions and is highly susceptible to local strains of *F. hepatica*. Because of the importance of both issues in the context of climate change and disease transmission, they are further discussed in the following section.

### High potential for invasion and spill-back of *F. hepatica* and other trematodes

Apart from the liver flukes *F. hepatica* and *F. gigantica* [21, 57], *O. viridis* has received limited attention regarding its potential to transmit other trematode parasites. However, some studies suggest that *O. viridis* and its sibling species, *Orientogalba ollula*, play a role as intermediate hosts of the human intestinal flukes *Echinostoma cinetorchis* (Echinostomatidae) in Korea [58] and of *Echinostoma revolutum* [59]. Our results demonstrated the natural infection (9%) of *O. viridis* from Spain with Trematoda, of which one had 98% similarity with *H. conoideum* (Echinostomatidae). The fully developed rediae of these species indicate the ability of *O. viridis* to participate in transmission within newly invaded areas. This species of echinostome has been found not only to infect a wide range of poultry (chickens, ducks and geese), causing significant economic losses, but also to display important zoonotic potential, as reported in Thailand [45]. Thus, veterinary authorities should seriously consider the surveillance of the potential spread of this species.

Additionally, we observed a high prevalence (86%) of naidid worms (*Chaetogaster* cf. *limnaei* (Annelida: Oligochaeta)) in the mantle cavity of *O. viridis*. Some authors suggest that this oligochaete relationship with snails ranges from mutualistic to parasitic, but it has a strong effect at both the snail population and community levels [60]. According to Ibrahim [61], *C. limnaei* is a potential regulator of trematode larvae inside the snail and is particularly able to ingest cercariae of *F. hepatica* [62]. The effect of this oligochaete on parasitic helminth has been explored in different freshwater snails such as the thiarid *Tarebia granifera* [63], and particularly a three-way biotic interaction among *C. limnaei*, echinostomes and nematodes was observed inside the planorbid *Helisoma anceps* [64].

Our experimental life-history trait data show the major potential for colonization in *O. viridis*, especially at warmer temperatures. The net reproduction rate is three to fourfold greater between the first and second reproduction peaks under both conditions and is particularly high at the end of reproductive age in tropical settings. There seems to be a reproductive trade-off with life expectancy, with individuals living longer and reaching sexual maturity later in temperate conditions. Globally, populations kept under both experimental conditions and subjected to *F. hepatica* infection had a significantly lower life expectancy than those not exposed to *F. hepatica* infection. However, cercariae shedding occurs well before suggesting a maintained epidemiological risk of this species of lymnaeid in the introduced areas. We did not test reproductive traits on exposed individuals; however, this will be done in the near future to explore the possible effects on sexual activity and population growth of *F. hepatica* on *O. viridis*, such as parasitological castration. Several studies explored the direct effect of trematode infections on the life-history traits of their snail hosts evidencing shifts on fecundity and growth (see for review [65]). Particularly in the case of lymnaeid snails, lower survival, fecundity rate and intrinsic increase rate were observed in *F. hepatica* exposed *Galba cubensis* [66].

The fact that individuals kept under both experimental conditions originate from the same field population is interesting because patchy field populations are maintained in cooler regions (i.e., southern Europe) but can undergo demographic explosions if the temperature rises in the summer. Korean *O. viridis* reach sexual maturity at approximately 3 weeks of age under laboratory conditions and reach a maximum height at 5 weeks of age [67]. It is worth noting that the native range of this species is Australasian and is well established in tropical (southeast Asia) and temperate Asia (Korean Peninsula and China) [20]. In Europe, the most common pond snail is the amphibious lymnaeid *Galba truncatula* [68, 69] but fully aquatic lymnaeids (e.g., *Stagnicola* spp., *Ampullaceana balthica*, *Peregriana peregra*) show an overspread of scattered populations in relation to the availability of suitable water bodies [69]. In the highly probable scenario of shifting temperatures towards slightly warmer conditions in southern Europe [70], *O. viridis* has the potential to initially colonize Mediterranean regions and possibly northern areas if local species fail to thrive and regress upwards.

If the above-pictured scenario occurs, eventual vacant niches due to global changes favouring *O. viridis* in Europe are notably important in disease epidemiology. For instance, *O. viridis* has been described as a major host of *Fasciola* spp. elsewhere [71]; thus, we tested its compatibility with different isolates of *F. hepatica* from Europe (Corsica) and the Caribbean (Cuba). The observed differences in survivorship, however, might be related to the particular virulence of the Corsican *F. hepatica* isolate towards the tested population of *O. viridis*. However, as expected, individuals reared in tropical and temperate settings showed prevalence values above 70% for both parasite isolates, indicating the high susceptibility of the parasite to the established *O. viridis* haplotype in Europe. Higher temperatures were consistent with higher values of prevalence and parasite intensity, providing clues for probable epidemiological scenarios under a warming climate. We also show that individuals of *O. viridis* are able to produce a very large number of metacercariae (over 900) starting very early after exposure in tropical conditions (only 23 d.p.e.), which directly attests to their transmission potential. Previously, several authors have explored metacercariae production in *G. truncatula* fed a high-quality diet and reported a mean number of shed cercariae of 359 ± 182 [72]. Similarly, a study using the same species [73] observed a range of 92–265 total metacercariae per shedding snail. In our experiment using *O. viridis* with an allopatric isolate of *F. hepatica*, these numbers nearly tripled, but we should also acknowledge that *O. viridis* usually grows larger than *G. truncatula*. In any case, our results are consistent with the reported values for experimental infections of *O. viridis* using a sympatric combination of Korean *F. hepatica* and *O. viridis* [57]. Taken together, our results demonstrate the potential for this exotic species to spread in the region and to be incorporated into the field transmission of fasciolosis in Europe, which is currently mainly caused by *G. truncatula*. The same outcome is expected if the species reaches farther tropical areas, such as the Caribbean basin, where fasciolosis transmission is endemic and driven by the local amphibious lymnaeid *Galba cubensis* [41].

We should note that this is not the first example of an invading lymnaeid snail in the open fields of Europe menacing to boost zoonotic diseases. Previously, the American native *P. columella* was first observed in France in 2004 [46] and is currently observed in artificial (Montpellier, [40]) and natural (Corsica, [39]) habitats. Previous studies have attempted to compare the metacercariae produced by *P. columella* and *G. truncatula* in France and have shown greater metacercariae production in the exotic (439 ± 118) snail host than in the native (264 ± 93) snail host [74]. Both recently introduced lymnaeid snails are capable of supporting a wide range of temperatures (cooler–warmer) and usually exhibit fully and semiaquatic behaviours ([18, 54]).

## Conclusions

Our experimental study shows the ability of the exotic species *O. viridis* to rapidly increase its population effectives, to perform in different climatic scenarios and to develop geographically different *F. hepatica* strains. Whether this species will succeed in Europe is unknown, but the odds are for a scenario in which a worldwide major snail host of *F. hepatica* occupy all available habitats of potential transmission foci, ravelling the epidemiology of fasciolosis. This research provides a comprehensive understanding of *O. viridis* biology, interactions with parasites and potential implications for disease transmission dynamics, offering valuable insights for further research and surveillance.

### Supplementary Information

Additional file 1: Dataset S1.

Additional file 2: Dataset S2.

## Data Availability

The data that support the findings of this study are available from the corresponding author upon reasonable request.
